# *trans*-Chloridobis(4-methyl­pyridine-κ*N*)(4,4′,4′′-tri-*tert*-butyl-2,2′:6′,2′′-terpyridine-κ^3^ *N*,*N*′,*N*′′)ruthenium(II) hexa­fluoridophosphate acetone monosolvate

**DOI:** 10.1107/S1600536812005594

**Published:** 2012-02-17

**Authors:** Christopher Redford, Carolina Gimbert-Suriñach, Mohan Bhadbhade, Stephen B. Colbran

**Affiliations:** a School of Chemistry, University of New South Wales, Sydney, 2052 NSW, Australia; b Mark Wainwright Analytical Centre, University of New South Wales, Sydney, 2052 NSW, Australia

## Abstract

The title compound, [RuCl(C_6_H_7_N)_2_(C_27_H_35_N_3_)]PF_6_·C_3_H_6_O, was obtained unintentionally as the product of the reaction of 1,1′-methyl­enebis(4-methyl­pyridinium) hexa­fluoriso­phos­phate and RuCl_3_(tpy*) (tpy* is 4,4′,4′′-tri-*tert*-butyl-2,2′:6′,2′′-terpyridine) in the presence of triethyl­amine and LiCl. The mol­ecular structure of the complex displays an octa­hedral geometry around the Ru^II^ ion and the unit cell contains an acetone solvent mol­ecule and one orientationally disordered PF_6_^−^ anion (occupancy ratio 0.75:0.25) which is hydrogen bonded to two H atoms of the tpy* ligand of the nearest [RuCl(pic)_2_(tpy*)]^+^ cation (pic is 4-methyl­pyridine). One of the *tert*-butyl groups of the tpy* ligand is also disordered over two sets of sites in a 0.75:0.25 ratio.

## Related literature

For details of the synthesis and properties of related ruthenium compounds containing a similar coordination environment, see: Suen *et al.* (1989 ▶); Coe *et al.* (1995 ▶); Tseng *et al.* (2008 ▶); Wasylenko *et al.* (2010 ▶); Duan *et al.* (2011 ▶).
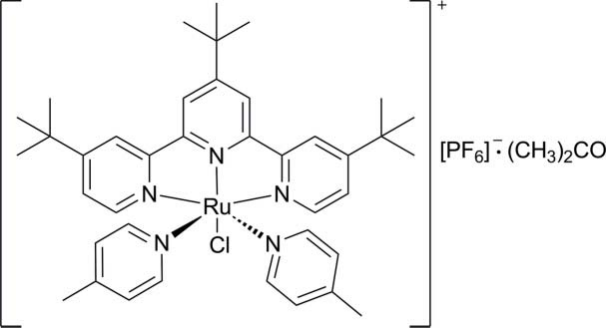


## Experimental

### 

#### Crystal data

[RuCl(C_6_H_7_N)_2_(C_27_H_35_N_3_)]PF_6_·C_3_H_6_O
*M* *_r_* = 927.40
Monoclinic, 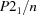

*a* = 16.4041 (9) Å
*b* = 12.7834 (6) Å
*c* = 21.9468 (11) Å
β = 107.865 (3)°
*V* = 4380.3 (4) Å^3^
*Z* = 4
Mo *K*α radiation
μ = 0.52 mm^−1^
*T* = 156 K
0.16 × 0.10 × 0.04 mm

#### Data collection

Bruker Kappa APEXII CCD diffractometer
Absorption correction: multi-scan (*SADABS*; Bruker, 2001 ▶) *T*_min_ = 0.920, *T*_max_ = 0.979
31904 measured reflections
7705 independent reflections
4203 reflections with *I* > 2σ(*I*)
*R*_int_ = 0.129

#### Refinement

*R*[*F* ^2^ > 2σ(*F* ^2^)] = 0.059
*wR*(*F* ^2^) = 0.171
*S* = 0.85
7705 reflections
566 parameters
81 restraints
H-atom parameters constrained
Δρ_max_ = 0.45 e Å^−3^
Δρ_min_ = −0.45 e Å^−3^

### 

Data collection: *APEX2* (Bruker, 2007 ▶); cell refinement: *APEX2*; data reduction: *APEX2*; program(s) used to solve structure: *SHELXS97* (Sheldrick, 2008 ▶); program(s) used to refine structure: *SHELXL97* (Sheldrick, 2008 ▶); molecular graphics: *SHELXTL-Plus* (Sheldrick, 2008 ▶); software used to prepare material for publication: *SHELXTL-Plus* and local programs.

## Supplementary Material

Crystal structure: contains datablock(s) I, global. DOI: 10.1107/S1600536812005594/hg5162sup1.cif

Structure factors: contains datablock(s) I. DOI: 10.1107/S1600536812005594/hg5162Isup2.hkl

Additional supplementary materials:  crystallographic information; 3D view; checkCIF report

## Figures and Tables

**Fig. 1.**
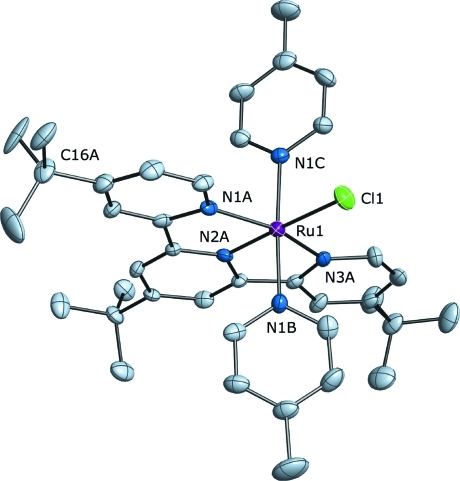
View of the [RuCl(pic)2(tpy*)]+ cation showing 50% thermal ellipsoids at 156 K (H-atoms are omitted for clarity; the labelled tert-butyl group C16A is rotationally disordered, and only the major orientation is shown).

**Fig. 2.**
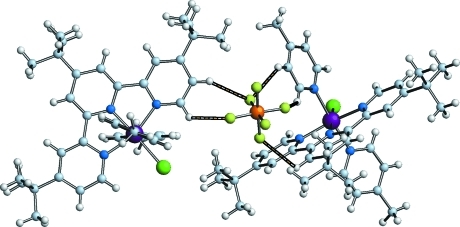
Ball-and-stick view of the crystal structure of [RuCl(pic)2(tpy*)][PF6] with the closest inter-ion C–H···F–P interactions, which range 2.49–2.79 Å, highlighted.

## supplementary crystallographic information

### Comment

Ruthenium(II) complexes containing terpyridine and pyridine ligands have recently been recognized as efficient water oxidation catalysts (Duan *et al.* 2011, Wasylenko *et al.* 2010 and Tseng *et al.* 2008). Most of the reported complexes contain bipyridine ligands and have the general formula [Ru(bpy)(tpy)L][PF_6_]_n_ (bpy = bipyridine; tpy = terpyridine; L = ligand; n = 1 or 2), but also the *cis* and *trans* isomers of complexes with pyridine ligands, analogous to the reported compound [RuCl(pic)_2_(tpy*)][PF_6_](pic = 4-picoline; tpy* = 4,4',4''-tri-*tert*-butyl-2,2':6',2''-terpyridine), have shown remarkable catalytic activity (Duan *et al.* 2011). The synthesis and modification of this kind of compounds have been studied for a long time (*e.g.* Suen *et al.* 1989 and Coe *et al.* 1995).

The octahedral geometry of the complex [RuCl(pic)_2_(tpy*)][PF_6_].(CH_3_)_2_CO has an average N—Ru—N angle of 87.9 (10) ° and an average N—Ru—Cl angle of 94.5 (15) °. The H···F distances of the hydrogen bonding between [PF_6_]^-^ anion and the hydrogen atoms of the terpyridine ligand are in the range of 2.4–2.5 Å. Careful inspection of the terpyridine ligand shows that the ligand does not lie on a single plane but its pyridyl rings are slightly twisted with respect to each other with an inter-ring dihedral angles range of 175.6 (6)–179.8 (6)°.

### Experimental

A mixture of RuCl_3_(tpy*) (25 mg, 0.041 mmol), 1,1'-methylenebis(4-methylpyridinium) hexafluorophosphate (20 mg, 0.041 mmol), LiCl (50 mg, 1.2 mmol), and NEt_3_ (0.1 ml, 1.4 mmol) was stirred in H_2_O/EtOH (1/1) (5 ml) at reflux for 18 h. After cooling, the solvent was removed under reduced pressure. The crude solid was then redissolved in hot toluene (20 ml). A red insoluble compound was discarded. The solvent was then removed, and the resulting purple solid was recrystallized by slow evaporation of an acetone/cyclohexane (9/1) mixture. Purple crystalline plates of [RuCl(pic)_2_(tpy*)][PF_6_].(CH_3_)_2_CO were obtained (19.9 mg, 0.0230 mmol, 56%). ^1^H NMR ((CD_3_)_2_CO, 300 MHz): δ 9.18–9.22 (d, 2H, *J* = 6.0 Hz, Ar—H), 8.75–8.79 (s, 2H, Ar—H), 8.69–8.63 (s, 2H, Ar—H), 7.95–8.00 (dd, *J*^3^ = 6.0 Hz, *J*^4^ = 1.3 Hz, 4H, Arpy*-H), 7.88–7.93 (dd, *J*^3^ = 6.0 Hz, *J*^4^ = 2.0 Hz, 2H, Artrpy*-H), 6.92–6.95 (d, *J* = 6.0 Hz, 4H, Ar—H), 2.15–2.19 (s, 6H, py-CH_3_), 1.51–1.56 (s, 9H, *tert*-but), 1.42–1.47 (s, 18H, *tert*-but). ^13^C NMR (((CD_3_)_2_CO, 300 MHz): δ 162, 161, 159, 156, 152, 151, 150, 149, 125, 120, 121, 35, 36, 30, 20.

### Refinement

The [PF_6_]^-^ anion and one of the butyl groups exhibited two fold orientational disorders. The major sites were refined anisotropically in each case, and appropriate constraints were applied (SADI, DELU) to maintain the geometry and displacement parameters of these entities. All H-atoms were positioned geometrically [C—H = 0.95 to 0.99 Å] and were refined using a riding-model approximation, with *U*_iso_(H) = 1.2 *U*_eq_(C) or 1.5 *U*_eq_(C_methyl_).

### Crystal data

**Table:**

[RuCl(C_6_H_7_N)_2_(C_27_H_35_N_3_)]PF_6_·C_3_H_6_O	*F*(000) = 1920
*M**_r_* = 927.40	*D*_x_ = 1.406 Mg m^−^^3^
Monoclinic, *P*2_1_/*n*	Mo *K*α radiation, λ = 0.71073 Å
Hall symbol: -P 2yn	Cell parameters from 1716 reflections
*a* = 16.4041 (9) Å	θ = 2.5–20.2°
*b* = 12.7834 (6) Å	µ = 0.52 mm^−^^1^
*c* = 21.9468 (11) Å	*T* = 156 K
β = 107.865 (3)°	Plate, yellow–brown
*V* = 4380.3 (4) Å^3^	0.16 × 0.10 × 0.04 mm
*Z* = 4	

### Data collection

**Table:**

Bruker Kappa APEXII CCD diffractometer	7705 independent reflections
Radiation source: fine-focus sealed tube	4203 reflections with *I* > 2σ(*I*)
Graphite monochromator	*R*_int_ = 0.129
φ scans, and ω scans with κ offsets	θ_max_ = 25.0°, θ_min_ = 2.4°
Absorption correction: multi-scan (*SADABS*; Bruker, 2001)	*h* = −19→19
*T*_min_ = 0.920, *T*_max_ = 0.979	*k* = −10→15
31904 measured reflections	*l* = −26→26

### Refinement

**Table:**

Refinement on *F*^2^	Primary atom site location: structure-invariant direct methods
Least-squares matrix: full	Secondary atom site location: difference Fourier map
*R*[*F*^2^ > 2σ(*F*^2^)] = 0.059	Hydrogen site location: inferred from neighbouring sites
*wR*(*F*^2^) = 0.171	H-atom parameters constrained
*S* = 0.85	*w* = 1/[σ^2^(*F*_o_^2^) + (0.1*P*)^2^ + 0.7175*P*] where *P* = (*F*_o_^2^ + 2*F*_c_^2^)/3
7705 reflections	(Δ/σ)_max_ = 0.001
566 parameters	Δρ_max_ = 0.45 e Å^−^^3^
81 restraints	Δρ_min_ = −0.45 e Å^−^^3^

### Special details

**Table:**

Geometry. All e.s.d.'s (except the e.s.d. in the dihedral angle between two l.s. planes) are estimated using the full covariance matrix. The cell e.s.d.'s are taken into account individually in the estimation of e.s.d.'s in distances, angles and torsion angles; correlations between e.s.d.'s in cell parameters are only used when they are defined by crystal symmetry. An approximate (isotropic) treatment of cell e.s.d.'s is used for estimating e.s.d.'s involving l.s. planes.
Refinement. Refinement of *F*^2^ against ALL reflections. The weighted *R*-factor *wR* and goodness of fit *S* are based on *F*^2^, conventional *R*-factors *R* are based on *F*, with *F* set to zero for negative *F*^2^. The threshold expression of *F*^2^ > σ(*F*^2^) is used only for calculating *R*-factors(gt) *etc*. and is not relevant to the choice of reflections for refinement. *R*-factors based on *F*^2^ are statistically about twice as large as those based on *F*, and *R*- factors based on ALL data will be even larger.

### Fractional atomic coordinates and isotropic or equivalent isotropic displacement parameters (Å^2^)

**Table:**

	*x*	*y*	*z*	*U*_iso_*/*U*_eq_	Occ. (<1)
Ru1	0.71663 (3)	0.19013 (4)	0.83778 (2)	0.01949 (17)	
N1A	0.6955 (3)	0.1972 (4)	0.7408 (2)	0.0188 (12)	
N2A	0.6460 (3)	0.0670 (4)	0.8110 (2)	0.0193 (12)	
N3A	0.7108 (3)	0.1373 (4)	0.9254 (2)	0.0200 (12)	
C1A	0.7264 (4)	0.2657 (5)	0.7070 (3)	0.0232 (15)	
H1A	0.7629	0.3199	0.7297	0.028*	
C2A	0.7084 (4)	0.2620 (5)	0.6419 (3)	0.0284 (17)	
H2A	0.7324	0.3124	0.6206	0.034*	
C3A	0.6543 (4)	0.1835 (5)	0.6067 (3)	0.0277 (16)	
C4A	0.6230 (4)	0.1128 (5)	0.6419 (3)	0.0228 (15)	
H4A	0.5861	0.0582	0.6201	0.027*	
C5A	0.6436 (4)	0.1194 (5)	0.7069 (3)	0.0207 (15)	
C6A	0.6163 (4)	0.0428 (5)	0.7478 (3)	0.0168 (14)	
C7A	0.5691 (4)	−0.0465 (5)	0.7282 (3)	0.0193 (15)	
H7A	0.5486	−0.0625	0.6838	0.023*	
C8A	0.5508 (4)	−0.1138 (5)	0.7717 (3)	0.0193 (15)	
C9A	0.5825 (4)	−0.0854 (5)	0.8367 (3)	0.0235 (16)	
H9A	0.5710	−0.1291	0.8681	0.028*	
C10A	0.6295 (4)	0.0038 (5)	0.8554 (3)	0.0169 (14)	
C11A	0.6657 (4)	0.0454 (5)	0.9205 (3)	0.0195 (15)	
C12A	0.6569 (4)	−0.0026 (5)	0.9753 (3)	0.0268 (16)	
H12A	0.6278	−0.0677	0.9712	0.032*	
C13A	0.6894 (4)	0.0421 (5)	1.0354 (3)	0.0244 (16)	
C14A	0.7324 (4)	0.1373 (5)	1.0382 (3)	0.0283 (17)	
H14A	0.7552	0.1719	1.0781	0.034*	
C15A	0.7419 (4)	0.1815 (5)	0.9835 (3)	0.0259 (15)	
H15A	0.7717	0.2460	0.9870	0.031*	
C16A	0.6317 (4)	0.1744 (5)	0.5347 (3)	0.0322 (18)	
C171	0.6684 (6)	0.2656 (7)	0.5057 (4)	0.042 (3)	0.75
H17A	0.6485	0.3321	0.5183	0.062*	0.75
H17B	0.6491	0.2597	0.4589	0.062*	0.75
H17C	0.7312	0.2632	0.5214	0.062*	0.75
C181	0.6659 (8)	0.0728 (7)	0.5174 (5)	0.067 (4)	0.75
H18A	0.7274	0.0677	0.5401	0.100*	0.75
H18B	0.6567	0.0706	0.4711	0.100*	0.75
H18C	0.6360	0.0140	0.5297	0.100*	0.75
C191	0.5336 (6)	0.1779 (9)	0.5060 (4)	0.055 (3)	0.75
H19A	0.5084	0.1184	0.5220	0.082*	0.75
H19B	0.5179	0.1742	0.4592	0.082*	0.75
H19C	0.5119	0.2433	0.5185	0.082*	0.75
C172	0.609 (2)	0.2813 (14)	0.5044 (17)	0.072 (13)*	0.25
H17D	0.5523	0.3019	0.5060	0.107*	0.25
H17E	0.6087	0.2786	0.4596	0.107*	0.25
H17F	0.6516	0.3326	0.5278	0.107*	0.25
C182	0.7120 (14)	0.133 (2)	0.5209 (18)	0.072 (12)*	0.25
H18D	0.7616	0.1755	0.5444	0.108*	0.25
H18E	0.7041	0.1383	0.4749	0.108*	0.25
H18F	0.7215	0.0602	0.5345	0.108*	0.25
C192	0.5585 (15)	0.098 (2)	0.5047 (15)	0.057 (10)*	0.25
H19D	0.5805	0.0258	0.5109	0.086*	0.25
H19E	0.5347	0.1120	0.4588	0.086*	0.25
H19F	0.5136	0.1058	0.5252	0.086*	0.25
C20A	0.4974 (4)	−0.2128 (5)	0.7520 (3)	0.0262 (16)	
C21A	0.4683 (5)	−0.2287 (5)	0.6793 (3)	0.0378 (19)	
H21A	0.4321	−0.1698	0.6586	0.057*	
H21B	0.4356	−0.2939	0.6687	0.057*	
H21C	0.5186	−0.2326	0.6642	0.057*	
C22A	0.5525 (5)	−0.3069 (5)	0.7826 (4)	0.0385 (19)	
H22A	0.6025	−0.3108	0.7672	0.058*	
H22B	0.5187	−0.3711	0.7709	0.058*	
H22C	0.5715	−0.2990	0.8293	0.058*	
C23A	0.4178 (4)	−0.2056 (5)	0.7740 (3)	0.0339 (18)	
H23A	0.4351	−0.2041	0.8209	0.051*	
H23B	0.3812	−0.2666	0.7583	0.051*	
H23C	0.3862	−0.1416	0.7569	0.051*	
C24A	0.6783 (5)	−0.0119 (6)	1.0949 (3)	0.0334 (18)	
C25A	0.7219 (6)	−0.1177 (6)	1.1023 (4)	0.053 (2)	
H25A	0.7141	−0.1540	1.1395	0.079*	
H25B	0.7832	−0.1081	1.1086	0.079*	
H25C	0.6966	−0.1596	1.0637	0.079*	
C26A	0.5839 (5)	−0.0252 (7)	1.0869 (3)	0.045 (2)	
H26A	0.5576	−0.0693	1.0496	0.067*	
H26B	0.5560	0.0435	1.0808	0.067*	
H26C	0.5770	−0.0583	1.1253	0.067*	
C27A	0.7198 (5)	0.0517 (7)	1.1563 (3)	0.049 (2)	
H27A	0.6946	0.1220	1.1516	0.074*	
H27B	0.7816	0.0572	1.1633	0.074*	
H27C	0.7095	0.0166	1.1929	0.074*	
N1B	0.8248 (3)	0.0994 (4)	0.8493 (2)	0.0220 (13)	
C1B	0.8339 (4)	0.0286 (5)	0.8059 (3)	0.0264 (16)	
H1B	0.7881	0.0204	0.7673	0.032*	
C2B	0.9059 (5)	−0.0320 (5)	0.8148 (3)	0.0320 (17)	
H2B	0.9095	−0.0785	0.7820	0.038*	
C3B	0.9721 (4)	−0.0261 (6)	0.8702 (3)	0.0311 (17)	
C4B	0.9635 (5)	0.0463 (6)	0.9149 (3)	0.0374 (19)	
H4B	1.0084	0.0541	0.9541	0.045*	
C5B	0.8908 (4)	0.1074 (5)	0.9035 (3)	0.0298 (17)	
H5B	0.8874	0.1567	0.9351	0.036*	
C6B	1.0506 (5)	−0.0932 (7)	0.8829 (4)	0.062 (3)	
H6B1	1.0342	−0.1671	0.8819	0.093*	
H6B2	1.0903	−0.0762	0.9251	0.093*	
H6B3	1.0785	−0.0802	0.8500	0.093*	
N1C	0.6102 (3)	0.2869 (4)	0.8311 (2)	0.0218 (13)	
C1C	0.6196 (4)	0.3761 (5)	0.8650 (3)	0.0282 (17)	
H1C	0.6747	0.3936	0.8929	0.034*	
C2C	0.5518 (5)	0.4435 (5)	0.8606 (3)	0.0350 (19)	
H2C	0.5619	0.5071	0.8841	0.042*	
C3C	0.4695 (5)	0.4195 (6)	0.8226 (3)	0.0339 (18)	
C4C	0.4608 (4)	0.3255 (6)	0.7891 (3)	0.0346 (18)	
H4C	0.4059	0.3043	0.7626	0.042*	
C5C	0.5306 (4)	0.2629 (5)	0.7940 (3)	0.0276 (16)	
H5C	0.5223	0.1998	0.7700	0.033*	
C6C	0.3960 (5)	0.4923 (6)	0.8152 (4)	0.047 (2)	
H6C1	0.4136	0.5495	0.8462	0.071*	
H6C2	0.3482	0.4542	0.8229	0.071*	
H6C3	0.3778	0.5210	0.7717	0.071*	
Cl1	0.81303 (11)	0.34102 (13)	0.86712 (8)	0.0306 (4)	
O1AC	0.5718 (4)	0.7458 (5)	−0.0482 (3)	0.0728 (19)	
C1AC	0.5987 (7)	0.6159 (8)	0.0311 (5)	0.085 (3)	
H1A1	0.6530	0.6086	0.0216	0.128*	
H1A2	0.5691	0.5483	0.0252	0.128*	
H1A3	0.6099	0.6393	0.0754	0.128*	
C2AC	0.5443 (6)	0.6938 (7)	−0.0127 (4)	0.054 (2)	
C3AC	0.4551 (7)	0.7086 (8)	−0.0114 (6)	0.102 (4)	
H3A1	0.4300	0.7700	−0.0371	0.153*	
H3A2	0.4554	0.7193	0.0329	0.153*	
H3A3	0.4211	0.6465	−0.0290	0.153*	
F1B	0.9240 (12)	0.389 (3)	0.1759 (19)	0.155 (13)*	0.25
F2B	0.831 (2)	0.5194 (15)	0.1385 (17)	0.116 (13)*	0.25
F3B	0.816 (2)	0.2843 (15)	0.1650 (14)	0.096 (11)*	0.25
F4B	0.7297 (13)	0.376 (3)	0.1182 (18)	0.129 (12)*	0.25
F5B	0.815 (2)	0.421 (3)	0.2162 (11)	0.119 (10)*	0.25
F6B	0.832 (2)	0.365 (2)	0.0845 (11)	0.111 (10)*	0.25
F1A	0.9018 (4)	0.4510 (6)	0.1285 (3)	0.0741 (19)	0.75
F2A	0.7893 (5)	0.5195 (4)	0.1500 (4)	0.069 (2)	0.75
F3A	0.8619 (5)	0.2875 (5)	0.1534 (3)	0.067 (2)	0.75
F4A	0.7523 (5)	0.3622 (6)	0.1762 (5)	0.094 (3)	0.75
F5A	0.8864 (5)	0.4190 (5)	0.2235 (3)	0.0580 (17)	0.75
F6A	0.7704 (6)	0.3909 (5)	0.0781 (3)	0.080 (2)	0.75
P1	0.82639 (14)	0.40259 (16)	0.15067 (11)	0.0457 (6)	

### Atomic displacement parameters (Å^2^)

**Table:**

	*U*^11^	*U*^22^	*U*^33^	*U*^12^	*U*^13^	*U*^23^
Ru1	0.0200 (3)	0.0179 (3)	0.0194 (3)	−0.0006 (3)	0.0043 (2)	−0.0013 (3)
N1A	0.017 (3)	0.013 (3)	0.024 (3)	−0.002 (2)	0.002 (2)	0.000 (2)
N2A	0.023 (3)	0.015 (3)	0.020 (3)	0.006 (2)	0.007 (2)	0.001 (2)
N3A	0.012 (3)	0.024 (3)	0.020 (3)	0.003 (2)	0.000 (2)	−0.005 (2)
C1A	0.022 (4)	0.018 (4)	0.028 (4)	0.000 (3)	0.005 (3)	0.007 (3)
C2A	0.030 (4)	0.023 (4)	0.033 (4)	−0.001 (3)	0.010 (3)	0.011 (3)
C3A	0.032 (4)	0.029 (4)	0.022 (3)	0.005 (4)	0.007 (3)	0.007 (3)
C4A	0.022 (4)	0.024 (4)	0.018 (3)	0.002 (3)	0.001 (3)	0.003 (3)
C5A	0.014 (4)	0.018 (4)	0.028 (4)	0.004 (3)	0.003 (3)	0.004 (3)
C6A	0.019 (4)	0.014 (3)	0.016 (3)	0.002 (3)	0.004 (3)	0.002 (3)
C7A	0.018 (4)	0.024 (4)	0.014 (3)	0.000 (3)	0.002 (3)	0.001 (3)
C8A	0.024 (4)	0.011 (3)	0.022 (4)	0.002 (3)	0.006 (3)	0.001 (3)
C9A	0.026 (4)	0.025 (4)	0.024 (4)	0.002 (3)	0.014 (3)	0.006 (3)
C10A	0.019 (4)	0.016 (3)	0.015 (3)	0.002 (3)	0.005 (3)	0.005 (3)
C11A	0.015 (4)	0.023 (4)	0.020 (3)	0.005 (3)	0.004 (3)	0.004 (3)
C12A	0.019 (4)	0.042 (4)	0.019 (4)	0.006 (3)	0.005 (3)	0.004 (3)
C13A	0.023 (4)	0.033 (4)	0.018 (4)	0.012 (3)	0.007 (3)	0.004 (3)
C14A	0.025 (4)	0.037 (4)	0.021 (4)	0.005 (4)	0.005 (3)	−0.010 (3)
C15A	0.028 (4)	0.028 (4)	0.021 (3)	−0.003 (3)	0.006 (3)	−0.007 (3)
C16A	0.043 (5)	0.035 (5)	0.019 (3)	−0.007 (4)	0.009 (3)	0.009 (3)
C171	0.039 (7)	0.059 (7)	0.022 (5)	−0.004 (6)	0.002 (5)	0.016 (5)
C181	0.142 (13)	0.041 (7)	0.033 (6)	−0.006 (8)	0.050 (8)	−0.009 (6)
C191	0.054 (8)	0.082 (9)	0.017 (5)	−0.026 (7)	−0.007 (5)	0.024 (6)
C20A	0.032 (4)	0.017 (4)	0.030 (4)	−0.004 (3)	0.011 (3)	−0.005 (3)
C21A	0.053 (5)	0.030 (4)	0.031 (4)	−0.018 (4)	0.014 (4)	−0.003 (3)
C22A	0.036 (5)	0.023 (4)	0.055 (5)	−0.004 (4)	0.011 (4)	−0.005 (4)
C23A	0.023 (4)	0.036 (5)	0.044 (4)	−0.004 (3)	0.012 (3)	−0.006 (4)
C24A	0.032 (4)	0.049 (5)	0.021 (4)	0.005 (4)	0.011 (3)	0.008 (3)
C25A	0.064 (6)	0.059 (6)	0.039 (5)	0.012 (5)	0.020 (4)	0.017 (4)
C26A	0.033 (5)	0.069 (6)	0.034 (4)	−0.002 (4)	0.012 (4)	0.005 (4)
C27A	0.043 (5)	0.083 (6)	0.022 (4)	−0.006 (5)	0.011 (4)	−0.001 (4)
N1B	0.021 (3)	0.025 (3)	0.018 (3)	0.001 (3)	0.004 (3)	−0.003 (2)
C1B	0.020 (4)	0.021 (4)	0.036 (4)	−0.002 (3)	0.005 (3)	0.005 (3)
C2B	0.034 (5)	0.029 (4)	0.037 (4)	0.010 (4)	0.017 (4)	0.006 (3)
C3B	0.025 (4)	0.040 (5)	0.032 (4)	0.010 (4)	0.014 (4)	0.008 (4)
C4B	0.022 (4)	0.055 (5)	0.031 (4)	0.007 (4)	0.002 (3)	0.005 (4)
C5B	0.024 (4)	0.038 (4)	0.030 (4)	−0.003 (3)	0.012 (3)	−0.004 (3)
C6B	0.034 (5)	0.079 (7)	0.069 (6)	0.030 (5)	0.012 (5)	0.015 (5)
N1C	0.023 (3)	0.020 (3)	0.022 (3)	0.000 (2)	0.006 (3)	0.003 (2)
C1C	0.027 (4)	0.024 (4)	0.030 (4)	0.004 (3)	0.003 (3)	0.000 (3)
C2C	0.049 (5)	0.022 (4)	0.035 (4)	0.006 (4)	0.015 (4)	−0.003 (3)
C3C	0.040 (5)	0.037 (5)	0.027 (4)	0.008 (4)	0.014 (4)	0.013 (4)
C4C	0.022 (4)	0.042 (5)	0.041 (4)	0.011 (4)	0.010 (3)	0.007 (4)
C5C	0.031 (5)	0.027 (4)	0.026 (4)	0.004 (3)	0.011 (3)	0.004 (3)
C6C	0.044 (5)	0.050 (5)	0.054 (5)	0.022 (4)	0.025 (4)	0.006 (4)
Cl1	0.0310 (10)	0.0252 (10)	0.0373 (10)	−0.0103 (8)	0.0129 (8)	−0.0084 (8)
O1AC	0.081 (5)	0.080 (5)	0.060 (4)	−0.012 (4)	0.025 (4)	0.033 (4)
C1AC	0.089 (9)	0.082 (8)	0.091 (8)	0.016 (7)	0.036 (7)	0.040 (6)
C2AC	0.071 (7)	0.042 (5)	0.055 (5)	0.003 (5)	0.030 (5)	0.006 (5)
C3AC	0.080 (9)	0.066 (8)	0.172 (13)	0.027 (6)	0.059 (8)	0.049 (7)
F1A	0.043 (4)	0.105 (6)	0.079 (5)	−0.014 (4)	0.025 (4)	−0.003 (4)
F2A	0.050 (5)	0.030 (3)	0.125 (7)	0.012 (3)	0.026 (5)	0.001 (3)
F3A	0.074 (6)	0.044 (4)	0.066 (5)	0.036 (3)	−0.003 (4)	−0.023 (3)
F4A	0.058 (5)	0.076 (5)	0.168 (8)	−0.001 (4)	0.066 (5)	0.020 (6)
F5A	0.072 (5)	0.052 (4)	0.042 (3)	−0.002 (3)	0.006 (3)	−0.014 (3)
F6A	0.075 (6)	0.062 (5)	0.063 (4)	−0.010 (4)	−0.039 (4)	−0.001 (3)
P1	0.0397 (14)	0.0356 (13)	0.0572 (14)	0.0029 (10)	0.0081 (11)	−0.0080 (11)

### Geometric parameters (Å, º)

**Table:**

Ru1—N2A	1.936 (5)	C22A—H22A	0.9800
Ru1—N1A	2.051 (5)	C22A—H22B	0.9800
Ru1—N3A	2.067 (5)	C22A—H22C	0.9800
Ru1—N1B	2.070 (5)	C23A—H23A	0.9800
Ru1—N1C	2.109 (5)	C23A—H23B	0.9800
Ru1—Cl1	2.4517 (17)	C23A—H23C	0.9800
N1A—C1A	1.343 (8)	C24A—C26A	1.513 (10)
N1A—C5A	1.370 (8)	C24A—C25A	1.516 (10)
N2A—C10A	1.357 (7)	C24A—C27A	1.542 (10)
N2A—C6A	1.357 (7)	C25A—H25A	0.9800
N3A—C15A	1.344 (7)	C25A—H25B	0.9800
N3A—C11A	1.375 (8)	C25A—H25C	0.9800
C1A—C2A	1.369 (9)	C26A—H26A	0.9800
C1A—H1A	0.9500	C26A—H26B	0.9800
C2A—C3A	1.404 (9)	C26A—H26C	0.9800
C2A—H2A	0.9500	C27A—H27A	0.9800
C3A—C4A	1.386 (9)	C27A—H27B	0.9800
C3A—C16A	1.513 (9)	C27A—H27C	0.9800
C4A—C5A	1.365 (8)	N1B—C5B	1.344 (8)
C4A—H4A	0.9500	N1B—C1B	1.355 (8)
C5A—C6A	1.487 (8)	C1B—C2B	1.376 (9)
C6A—C7A	1.372 (8)	C1B—H1B	0.9500
C7A—C8A	1.386 (8)	C2B—C3B	1.361 (9)
C7A—H7A	0.9500	C2B—H2B	0.9500
C8A—C9A	1.406 (8)	C3B—C4B	1.388 (10)
C8A—C20A	1.524 (8)	C3B—C6B	1.500 (10)
C9A—C10A	1.368 (8)	C4B—C5B	1.383 (9)
C9A—H9A	0.9500	C4B—H4B	0.9500
C10A—C11A	1.468 (8)	C5B—H5B	0.9500
C11A—C12A	1.395 (8)	C6B—H6B1	0.9800
C12A—C13A	1.386 (9)	C6B—H6B2	0.9800
C12A—H12A	0.9500	C6B—H6B3	0.9800
C13A—C14A	1.399 (9)	N1C—C1C	1.344 (8)
C13A—C24A	1.537 (9)	N1C—C5C	1.345 (8)
C14A—C15A	1.378 (9)	C1C—C2C	1.388 (9)
C14A—H14A	0.9500	C1C—H1C	0.9500
C15A—H15A	0.9500	C2C—C3C	1.386 (10)
C16A—C181	1.510 (10)	C2C—H2C	0.9500
C16A—C172	1.516 (14)	C3C—C4C	1.393 (10)
C16A—C182	1.532 (14)	C3C—C6C	1.493 (10)
C16A—C192	1.535 (14)	C4C—C5C	1.373 (9)
C16A—C171	1.538 (9)	C4C—H4C	0.9500
C16A—C191	1.539 (9)	C5C—H5C	0.9500
C171—H17A	0.9800	C6C—H6C1	0.9800
C171—H17B	0.9800	C6C—H6C2	0.9800
C171—H17C	0.9800	C6C—H6C3	0.9800
C181—H18A	0.9800	O1AC—C2AC	1.211 (9)
C181—H18B	0.9800	C1AC—C2AC	1.478 (12)
C181—H18C	0.9800	C1AC—H1A1	0.9800
C191—H19A	0.9800	C1AC—H1A2	0.9800
C191—H19B	0.9800	C1AC—H1A3	0.9800
C191—H19C	0.9800	C2AC—C3AC	1.485 (12)
C172—H17D	0.9800	C3AC—H3A1	0.9800
C172—H17E	0.9800	C3AC—H3A2	0.9800
C172—H17F	0.9800	C3AC—H3A3	0.9800
C182—H18D	0.9800	F1B—P1	1.535 (18)
C182—H18E	0.9800	F2B—P1	1.523 (18)
C182—H18F	0.9800	F3B—P1	1.565 (18)
C192—H19D	0.9800	F4B—P1	1.563 (18)
C192—H19E	0.9800	F5B—P1	1.522 (18)
C192—H19F	0.9800	F6B—P1	1.561 (18)
C20A—C23A	1.526 (9)	F1A—P1	1.587 (6)
C20A—C22A	1.530 (9)	F2A—P1	1.612 (6)
C20A—C21A	1.531 (9)	F3A—P1	1.577 (5)
C21A—H21A	0.9800	F4A—P1	1.573 (7)
C21A—H21B	0.9800	F5A—P1	1.613 (6)
C21A—H21C	0.9800	F6A—P1	1.583 (6)
			
N2A—Ru1—N1A	80.0 (2)	C24A—C25A—H25A	109.5
N2A—Ru1—N3A	79.8 (2)	C24A—C25A—H25B	109.5
N1A—Ru1—N3A	159.7 (2)	H25A—C25A—H25B	109.5
N2A—Ru1—N1B	89.6 (2)	C24A—C25A—H25C	109.5
N1A—Ru1—N1B	91.02 (19)	H25A—C25A—H25C	109.5
N3A—Ru1—N1B	89.16 (19)	H25B—C25A—H25C	109.5
N2A—Ru1—N1C	92.9 (2)	C24A—C26A—H26A	109.5
N1A—Ru1—N1C	91.66 (19)	C24A—C26A—H26B	109.5
N3A—Ru1—N1C	89.02 (19)	H26A—C26A—H26B	109.5
N1B—Ru1—N1C	176.6 (2)	C24A—C26A—H26C	109.5
N2A—Ru1—Cl1	175.67 (15)	H26A—C26A—H26C	109.5
N1A—Ru1—Cl1	97.36 (14)	H26B—C26A—H26C	109.5
N3A—Ru1—Cl1	102.88 (15)	C24A—C27A—H27A	109.5
N1B—Ru1—Cl1	87.08 (15)	C24A—C27A—H27B	109.5
N1C—Ru1—Cl1	90.58 (15)	H27A—C27A—H27B	109.5
C1A—N1A—C5A	116.8 (5)	C24A—C27A—H27C	109.5
C1A—N1A—Ru1	129.0 (4)	H27A—C27A—H27C	109.5
C5A—N1A—Ru1	114.2 (4)	H27B—C27A—H27C	109.5
C10A—N2A—C6A	120.7 (5)	C5B—N1B—C1B	116.2 (6)
C10A—N2A—Ru1	119.9 (4)	C5B—N1B—Ru1	119.5 (4)
C6A—N2A—Ru1	119.3 (4)	C1B—N1B—Ru1	124.2 (4)
C15A—N3A—C11A	118.1 (5)	N1B—C1B—C2B	123.3 (6)
C15A—N3A—Ru1	129.2 (4)	N1B—C1B—H1B	118.3
C11A—N3A—Ru1	112.6 (4)	C2B—C1B—H1B	118.3
N1A—C1A—C2A	123.8 (6)	C3B—C2B—C1B	120.8 (7)
N1A—C1A—H1A	118.1	C3B—C2B—H2B	119.6
C2A—C1A—H1A	118.1	C1B—C2B—H2B	119.6
C1A—C2A—C3A	119.8 (6)	C2B—C3B—C4B	116.2 (6)
C1A—C2A—H2A	120.1	C2B—C3B—C6B	122.6 (7)
C3A—C2A—H2A	120.1	C4B—C3B—C6B	121.2 (7)
C4A—C3A—C2A	116.1 (6)	C5B—C4B—C3B	121.2 (7)
C4A—C3A—C16A	121.2 (6)	C5B—C4B—H4B	119.4
C2A—C3A—C16A	122.7 (6)	C3B—C4B—H4B	119.4
C5A—C4A—C3A	121.8 (6)	N1B—C5B—C4B	122.2 (6)
C5A—C4A—H4A	119.1	N1B—C5B—H5B	118.9
C3A—C4A—H4A	119.1	C4B—C5B—H5B	118.9
C4A—C5A—N1A	121.7 (6)	C3B—C6B—H6B1	109.5
C4A—C5A—C6A	124.5 (6)	C3B—C6B—H6B2	109.5
N1A—C5A—C6A	113.8 (5)	H6B1—C6B—H6B2	109.5
N2A—C6A—C7A	120.1 (5)	C3B—C6B—H6B3	109.5
N2A—C6A—C5A	112.7 (5)	H6B1—C6B—H6B3	109.5
C7A—C6A—C5A	127.2 (5)	H6B2—C6B—H6B3	109.5
C6A—C7A—C8A	121.5 (5)	C1C—N1C—C5C	117.0 (6)
C6A—C7A—H7A	119.3	C1C—N1C—Ru1	120.4 (4)
C8A—C7A—H7A	119.3	C5C—N1C—Ru1	122.5 (4)
C7A—C8A—C9A	116.5 (6)	N1C—C1C—C2C	122.3 (6)
C7A—C8A—C20A	123.2 (5)	N1C—C1C—H1C	118.9
C9A—C8A—C20A	120.3 (5)	C2C—C1C—H1C	118.9
C10A—C9A—C8A	121.3 (6)	C3C—C2C—C1C	121.1 (7)
C10A—C9A—H9A	119.4	C3C—C2C—H2C	119.5
C8A—C9A—H9A	119.4	C1C—C2C—H2C	119.5
N2A—C10A—C9A	119.9 (5)	C2C—C3C—C4C	115.6 (7)
N2A—C10A—C11A	111.9 (5)	C2C—C3C—C6C	122.2 (7)
C9A—C10A—C11A	128.1 (5)	C4C—C3C—C6C	122.2 (7)
N3A—C11A—C12A	120.3 (6)	C5C—C4C—C3C	120.9 (7)
N3A—C11A—C10A	115.6 (5)	C5C—C4C—H4C	119.6
C12A—C11A—C10A	124.1 (6)	C3C—C4C—H4C	119.6
C13A—C12A—C11A	121.8 (7)	N1C—C5C—C4C	123.0 (6)
C13A—C12A—H12A	119.1	N1C—C5C—H5C	118.5
C11A—C12A—H12A	119.1	C4C—C5C—H5C	118.5
C12A—C13A—C14A	116.2 (6)	C3C—C6C—H6C1	109.5
C12A—C13A—C24A	120.8 (6)	C3C—C6C—H6C2	109.5
C14A—C13A—C24A	122.9 (6)	H6C1—C6C—H6C2	109.5
C15A—C14A—C13A	120.5 (6)	C3C—C6C—H6C3	109.5
C15A—C14A—H14A	119.7	H6C1—C6C—H6C3	109.5
C13A—C14A—H14A	119.7	H6C2—C6C—H6C3	109.5
N3A—C15A—C14A	122.9 (6)	C2AC—C1AC—H1A1	109.5
N3A—C15A—H15A	118.5	C2AC—C1AC—H1A2	109.5
C14A—C15A—H15A	118.5	H1A1—C1AC—H1A2	109.5
C181—C16A—C3A	109.7 (6)	C2AC—C1AC—H1A3	109.5
C181—C16A—C172	136.5 (15)	H1A1—C1AC—H1A3	109.5
C3A—C16A—C172	109.8 (15)	H1A2—C1AC—H1A3	109.5
C181—C16A—C182	41.1 (11)	O1AC—C2AC—C1AC	121.3 (9)
C3A—C16A—C182	106.5 (15)	O1AC—C2AC—C3AC	120.4 (9)
C172—C16A—C182	109.3 (12)	C1AC—C2AC—C3AC	118.3 (8)
C181—C16A—C192	69.1 (11)	C2AC—C3AC—H3A1	109.5
C3A—C16A—C192	114.0 (14)	C2AC—C3AC—H3A2	109.5
C172—C16A—C192	109.5 (12)	H3A1—C3AC—H3A2	109.5
C182—C16A—C192	107.6 (12)	C2AC—C3AC—H3A3	109.5
C181—C16A—C171	108.9 (6)	H3A1—C3AC—H3A3	109.5
C3A—C16A—C171	111.9 (6)	H3A2—C3AC—H3A3	109.5
C172—C16A—C171	37.7 (12)	F5B—P1—F2B	92.2 (18)
C182—C16A—C171	73.0 (12)	F5B—P1—F1B	95 (2)
C192—C16A—C171	131.4 (14)	F2B—P1—F1B	94 (2)
C181—C16A—C191	110.2 (7)	F5B—P1—F6B	170.4 (17)
C3A—C16A—C191	108.5 (6)	F2B—P1—F6B	97.0 (18)
C172—C16A—C191	73.1 (12)	F1B—P1—F6B	87.0 (19)
C182—C16A—C191	141.6 (15)	F5B—P1—F4B	93.8 (19)
C192—C16A—C191	42.3 (11)	F2B—P1—F4B	103.0 (19)
C171—C16A—C191	107.6 (6)	F1B—P1—F4B	160.3 (19)
C16A—C171—H17A	109.5	F6B—P1—F4B	81.5 (17)
C16A—C171—H17B	109.5	F5B—P1—F3B	84.7 (17)
C16A—C171—H17C	109.5	F2B—P1—F3B	175.5 (18)
C16A—C181—H18A	109.5	F1B—P1—F3B	89.4 (18)
C16A—C181—H18B	109.5	F6B—P1—F3B	85.9 (16)
C16A—C181—H18C	109.5	F4B—P1—F3B	74.0 (17)
C16A—C191—H19A	109.5	F5B—P1—F4A	52.1 (13)
C16A—C191—H19B	109.5	F2B—P1—F4A	117.8 (14)
C16A—C191—H19C	109.5	F1B—P1—F4A	132.1 (16)
C16A—C172—H17D	109.5	F6B—P1—F4A	120.2 (12)
C16A—C172—H17E	109.5	F4B—P1—F4A	46.0 (14)
H17D—C172—H17E	109.5	F3B—P1—F4A	57.7 (12)
C16A—C172—H17F	109.5	F5B—P1—F3A	105.0 (12)
H17D—C172—H17F	109.5	F2B—P1—F3A	152.3 (15)
H17E—C172—H17F	109.5	F1B—P1—F3A	63.4 (14)
C16A—C182—H18D	109.5	F6B—P1—F3A	67.5 (12)
C16A—C182—H18E	109.5	F4B—P1—F3A	97.4 (13)
H18D—C182—H18E	109.5	F3B—P1—F3A	32.2 (10)
C16A—C182—H18F	109.5	F4A—P1—F3A	89.8 (5)
H18D—C182—H18F	109.5	F5B—P1—F6A	140.0 (15)
H18E—C182—H18F	109.5	F2B—P1—F6A	87.7 (14)
C16A—C192—H19D	109.5	F1B—P1—F6A	124.9 (16)
C16A—C192—H19E	109.5	F6B—P1—F6A	38.4 (11)
H19D—C192—H19E	109.5	F4B—P1—F6A	47.7 (14)
C16A—C192—H19F	109.5	F3B—P1—F6A	92.5 (11)
H19D—C192—H19F	109.5	F4A—P1—F6A	93.1 (5)
H19E—C192—H19F	109.5	F3A—P1—F6A	92.5 (4)
C8A—C20A—C23A	109.4 (5)	F5B—P1—F1A	123.9 (15)
C8A—C20A—C22A	108.5 (5)	F2B—P1—F1A	58.7 (14)
C23A—C20A—C22A	110.8 (5)	F1B—P1—F1A	47.8 (14)
C8A—C20A—C21A	112.0 (5)	F6B—P1—F1A	63.9 (12)
C23A—C20A—C21A	108.3 (6)	F4B—P1—F1A	136.2 (16)
C22A—C20A—C21A	107.8 (5)	F3B—P1—F1A	125.7 (12)
C20A—C21A—H21A	109.5	F4A—P1—F1A	175.5 (5)
C20A—C21A—H21B	109.5	F3A—P1—F1A	93.6 (5)
H21A—C21A—H21B	109.5	F6A—P1—F1A	89.7 (5)
C20A—C21A—H21C	109.5	F5B—P1—F2A	73.2 (12)
H21A—C21A—H21C	109.5	F2B—P1—F2A	29.2 (13)
H21B—C21A—H21C	109.5	F1B—P1—F2A	117.0 (15)
C20A—C22A—H22A	109.5	F6B—P1—F2A	114.2 (12)
C20A—C22A—H22B	109.5	F4B—P1—F2A	82.4 (13)
H22A—C22A—H22B	109.5	F3B—P1—F2A	146.4 (12)
C20A—C22A—H22C	109.5	F4A—P1—F2A	88.7 (4)
H22A—C22A—H22C	109.5	F3A—P1—F2A	178.1 (5)
H22B—C22A—H22C	109.5	F6A—P1—F2A	88.7 (4)
C20A—C23A—H23A	109.5	F1A—P1—F2A	87.9 (4)
C20A—C23A—H23B	109.5	F5B—P1—F5A	41.9 (13)
H23A—C23A—H23B	109.5	F2B—P1—F5A	90.0 (14)
C20A—C23A—H23C	109.5	F1B—P1—F5A	53.5 (15)
H23A—C23A—H23C	109.5	F6B—P1—F5A	140.3 (13)
H23B—C23A—H23C	109.5	F4B—P1—F5A	134.8 (16)
C26A—C24A—C25A	110.2 (7)	F3B—P1—F5A	89.9 (11)
C26A—C24A—C13A	109.6 (5)	F4A—P1—F5A	89.6 (5)
C25A—C24A—C13A	108.5 (6)	F3A—P1—F5A	88.8 (3)
C26A—C24A—C27A	108.8 (6)	F6A—P1—F5A	177.0 (4)
C25A—C24A—C27A	108.0 (6)	F1A—P1—F5A	87.5 (4)
C13A—C24A—C27A	111.7 (6)	F2A—P1—F5A	90.1 (4)

## References

[bb1] Bruker (2001). *SADABS.* Bruker AXS Inc., Madison, Wisconsin, USA.

[bb2] Bruker (2007). *APEX2* Bruker AXS Inc., Madison, Wisconsin, USA.

[bb3] Coe, B. J., Thompson, D. W., Culbertson, C. T., Schoonover, J. R. & Meyer, T. J. (1995). *Inorg. Chem.* **34**, 3385–3395.

[bb4] Duan, L., Xu, Y., Tong, L. & Sun, L. (2011). *ChemSusChem*, **4**, 238–244. 10.1002/cssc.201000313 21328553

[bb5] Sheldrick, G. M. (2008). *Acta Cryst.* A**64**, 112–122. 10.1107/S0108767307043930 18156677

[bb6] Suen, H. F., Wilson, S. W., Pomerantz, M. & Walsh, J. L. (1989). *Inorg. Chem.* **28**, 786–791.

[bb7] Tseng, H.-W., Zong, R., Muckerman, J. T. & Thummel, R. (2008). *Inorg. Chem.* **47**, 11763–11773. 10.1021/ic8014817 19006384

[bb8] Wasylenko, D. J., Ganesamoorthy, C., Koivisto, B. D. & Berlinguette, C. P. (2010). *Eur. J. Inorg. Chem.* pp. 3135–3142. 10.1021/ic902024s 20131861

